# Interrelations of love of life with psychosocial factors in university students

**DOI:** 10.1192/j.eurpsy.2022.1766

**Published:** 2022-09-01

**Authors:** E. Nikolaev, S. Petunova

**Affiliations:** Ulianov Chuvash State University, Social And Clinical Psychology Department, Cheboksary, Russian Federation

**Keywords:** Love of life, psychosocial factors, university students

## Abstract

**Introduction:**

Love of life as a positive evaluation of one‘s own life plays an important role in developing a person’s positive outlook on their own wellbeing.

**Objectives:**

Our goal is to define the nature of interrelations between manifestations of love of life and some psychosocial factors of health in university students.

**Methods:**

Using the English version of the Love of Life Scale (Ahmed M. Abdel-Khalek) and the Sociocultural Health Questionnaire (E. Nikolaev), we carried out an online survey of 136 university students of both genders. A correlation analysis helped to define the interrelations.

**Results:**

We established that a high level of the overall rate of Love of Life, on the one hand, corresponds to high self-evaluation of a person’s health (r=.31, p<.05) and happiness (r=.47, p<.05). On the other hand, it correlates with a high level of anti-suicidal barrier (r=.20, p<.05) and low frequency of headaches (r=-.18, p<.05). Students’ desire for a long life, which would enable to achieve everything they have been dreaming of, correlates with low weight (r=-.18, p<.05). A low level of stress is connected with a greater feeling of love in life (r=-.22, p<.05) and its perception as something beautiful and fascinating (r=-.29, p<.05). Better understanding of life correlates with lower frequency of smoking (r=-.19, p<.05).

**Conclusions:**

A psychological construct of Love of life, due to its negative correlations with the health risk factors has a great positive potential for a personality development and their health. It can serve as a target for a psychological impact in interventions.

**Disclosure:**

No significant relationships.

